# Assessment of geriatric and clinical domains for development and validation of a novel nomogram to predict the prognosis of older patients with breast cancer: a real-world retrospective cohort study

**DOI:** 10.3389/fonc.2023.1250927

**Published:** 2023-11-28

**Authors:** Yan Lin, Yu Song, Ying Xu, Changjun Wang, Yali Xu, Xin Huang, Qiang Sun

**Affiliations:** Department of Breast Disease, Peking Union Medical College Hospital, Peking Union Medical College, Beijing, China

**Keywords:** breast cancer, older patients, nomogram, prognosis, geriatric assessment

## Abstract

**Background:**

Breast cancer is a relatively heterogeneous disease in the older population. Survival in older breast cancer patients is not only affected by tumor-related factors, but also by geriatric assessment domains. How tumor clinical factors and geriatric factors specifically affect the survival rate of older patients and how to combine these two factors to predict the risk of death in older patients with breast cancer remain clinical questions to be addressed.

**Method:**

We used the Peking Union Medical College Hospital database to identify older patients (≥65 years) who were newly diagnosed with breast cancer between January 2013 and December 2019. Of the 641 eligible patients, we retrospectively analyzed the clinical and geriatric data of 556 patients who formed our study population. The primary outcomes were overall survival (OS) and breast cancer-specific survival (BCSS). Univariate and multivariate Cox regression analyses were conducted to identify independent prognostic factors and construct a nomogram to predict the 1-, 3-, and 5-year survival rates. The performance of the constructed nomogram was evaluated using calibration curve, receiver operating characteristic (ROC) curve, and decision curve analysis (DCA).

**Results:**

Multivariate Cox regression analysis revealed seven independent prognostic factors associated with OS in older patients with breast cancer: age, tumor stage, lymph node stage, intrinsic molecular subtype, functional status, comorbidities, and psychological state. Nomogram based on these seven factors yielded excellent performance, with area under the ROC curve (AUROC) of 0.748. Similarly, the nomogram for BCSS had an AUROC of 0.760. Moreover, the calibration curve and DCA revealed good predictive accuracy between the actual and predicted probabilities.

**Conclusion:**

Independent prognostic factors for OS and BCSS in older patients with breast cancer in China were determined in our study. A novel nomogram for predicting the 1-, 3-, and 5-year OS and BCSS in this patient population was developed and validated. The nomogram exhibited good accuracy, indicating its potential for clinical decision making and improving outcomes.

## 1 Introduction

The incidence of breast cancer in the older population is increasing due to the progressive increase in the incidence of breast cancer and an increasingly aging society (1, 2). Breast cancers in older patients are characterized by less invasive subtypes. However, the risk of breast cancer-associated mortality is higher in older patients than in patients of other age groups with the same cancer subtype (3, 4). Therefore, healthcare professionals need accurate tools to identify the factors associated with mortality in older breast cancer patients and develop novel prognostic models to help guide clinical decision-making.

Our previous studies showed that both tumor-related and geriatric factors affect breast cancer survival in older patients (5). Some studies on solid tumors have reported similar findings, suggesting that three factors (functional status, comorbidities, and psychological state) have a significant impact on patient survival rate (6–9). However, the integration of geriatric and tumor-related factors and the development of a precise prognostic tool were the primary challenges (10).

Prognostic model involving geriatric and tumor-related domains for older patients with breast cancer is not yet available. To the best of our knowledge, this study is the first to analyze both geriatric (functional status, comorbidities, psychological state) and clinical (tumor stage, lymph node stage, intrinsic molecular subtype) information regarding survival in older patients with breast cancer. We conducted a real-world retrospective cohort study on older patients with early-stage breast cancer who received treatment at our medical center. Based on the results of previous studies on solid tumors in the older population (5, 6), we incorporated the abovementioned geriatric assessment (GA) and tumor-related domains into the survival analysis. Our main objective was to develop and validate a novel nomogram to predict the prognosis of older patients with breast cancer in China.

## 2 Patients and methods

### 2.1 Study population

The Peking Union Medical College Hospital (PUMCH) database has archived patient information since 1975, including age at diagnosis, functional status, comorbidities, psychological status, surgical methods, tumor histology, treatment history, recurrence/metastasis (local, regional, distant), and survival. To analyze associations between clinical information and mortality, key data were extracted from both the archive and the digital records of this study.

Older patients with breast cancer who were admitted to our center’s breast surgery department between January 2013 and December 2019 formed our study population. The inclusion criteria were: (i) patients who were ≥65 years of age, (ii) those with a preliminary clinical diagnosis of early breast cancer, (iii) those who were intended for surgical treatment, and (iv) those with no metastatic tumors in any organ. Patients who met the inclusion criteria were approached for an interview, and three geriatric details (functional status, comorbidity, and psychological state) were initially obtained. After the patients were admitted to the ward, the three components of geriatric assessment were verified again or supplemented. Moreover, the clinical information was also refined in the ward. Dedicated follow-up staff regularly collected survival information. Patients with complete information regarding the three components (GA, clinical information, and follow-up information) were included in our study.

The study was approved by the Institutional Review Board of Peking Union Medical College Hospital (ZS-2682). The principles of the Helsinki Declaration of 1964 and subsequent amendments, or comparable ethical norms, were adhered to.

### 2.2 GA domains

The GA method used in this study was developed on the basis of previous studies and reports based on similar questionnaires (5, 11–13). The comprehensive geriatric assessment (CGA) contains a large number of domains and a cumbersome questionnaire; therefore, the use of all CGA domains to assess older patients in surgical oncology is too time-consuming to be widely practiced (14). Therefore, we used functional status, comorbidity, and psychological state, which are the three domains most closely associated with survival in older patients assessed for geriatric solid tumors. The relevance of these three domains in survival of elderly patients with breast cancer has been previously confirmed (5).

The Eastern Cooperative Oncology Group Performance Status (ECOG PS) is currently the most commonly used functional domain in clinical practice due to its simplicity. However, studies have shown that activities of daily living (ADL) are more objective and accurate than ECOG (15). Nevertheless, the ADL questionnaire content is repetitive, and the score results are not easy to interpret. Therefore, based on the Barthel Index score, we simplified the ADL questionnaire including 10 items to measure ADL performance, and interpreted the results as normal or abnormal (16). The items that were assessed included continence and independence in a range of daily activities such as bathing, feeding, dressing, going to the bathroom, getting up and moving around the house. Each patient had a score from 0 to 100, which is a measure of the patient’s level of independence. Functional status was graded as abnormal (0-60) or normal (61-100) according to each patient’s status. If insufficient, the status was marked unknown.

Comorbidities were assessed based on the Charlson Comorbidity Index (CCI) score, which includes 17 comorbid conditions (myocardial infarction, chronic pulmonary disease, rheumatologic disease, congestive heart failure, peripheral vascular disease, cerebrovascular disease, dementia, peptic ulcer disease, liver disease, diabetes, hemiplegia or paraplegia, renal disease, any malignancy, lymphoma, leukemia, and human immunodeficiency virus infection) (17). Metastatic cancer, paraplegia and HIV infection were excluded due to a conflict of treatment principles, so that 14 co-morbid conditions were included in this study. For each comorbidity, 4 types of weights (1, 2, 3 and 6) were assigned based on the associated mortality risk. Two categories of comorbidity were used in this study: none to mild (CCI: 0-2) and severe (CCI: ≥3).

Psychological state was evaluated according to the updated Geriatric Depression Scale (GDS-15), which helps to evaluate depression in geriatric individuals with a collection of 15 factors (18). Based on the most common clinical criteria, psychological state was categorized as normal or non-depressive, abnormal or depressive state, and unknown.

### 2.3 Tumor-related domains

We also gathered and analyzed clinical information, such as tumor stage, lymph node stage, and intrinsic molecular subtypes. Data of all-cause mortality and breast cancer-specific mortality were obtained from our center and hospital databases, which have complete records of patient visits, medication information, and clinical referral records. Our center has a dedicated follow-up staff to capture this relevant information, and missing data were supplemented by telephone contact.

### 2.4 Statistical analysis

Categorical variables were expressed as numbers and percentages. Pearson’s chi-square test was used to compare the results of different groups. The primary endpoints were overall survival (OS) and breast cancer specific survival (BCSS). OS was calculated as the time from the date of cancer diagnosis until the last date of vital status. BCSS was defined as the time from diagnosis of breast cancer to the breast cancer-related death. A Cox proportional hazards model was constructed for univariate and multivariate analyses of OS and BCSS. To select the variables for the nomogram, multivariate Cox regression model and backward stepwise selection were adopted based on the Akaike information criterion. A nomogram was constructed to predict the 1-, 3-, and 5-year OS and BCSS. Survival Receiver Operating Characteristic (ROC) curve and C-index were calculated to evaluate the discriminatory ability of the nomogram. Visual inspection of calibration was conducted by calibration curves. The potential clinical utility of the nomogram was evaluated by the decision curve analysis (DCA). Due to the high mortality rate among the older population, we fitted competing risk models with breast cancer-specific death and non-breast cancer-specific death as competing events. The Fine and Gray method of competing risk model was used for univariate and multivariate analyses of BCSS. A nomogram based on competing risk model was constructed to predict 3- and 5-year BCSS. To evaluate the discriminatory ability of the nomogram, C-index was produced for each model. Calibration curves were used for visual inspection of calibration. Statistical analyses were performed using the R (4.2.2) software. All statistical tests were two-tailed, and a P value less than 0.05 is considered statistical significant.

## 3 Results

### 3.1 Patient characteristics

**Figure 1:**
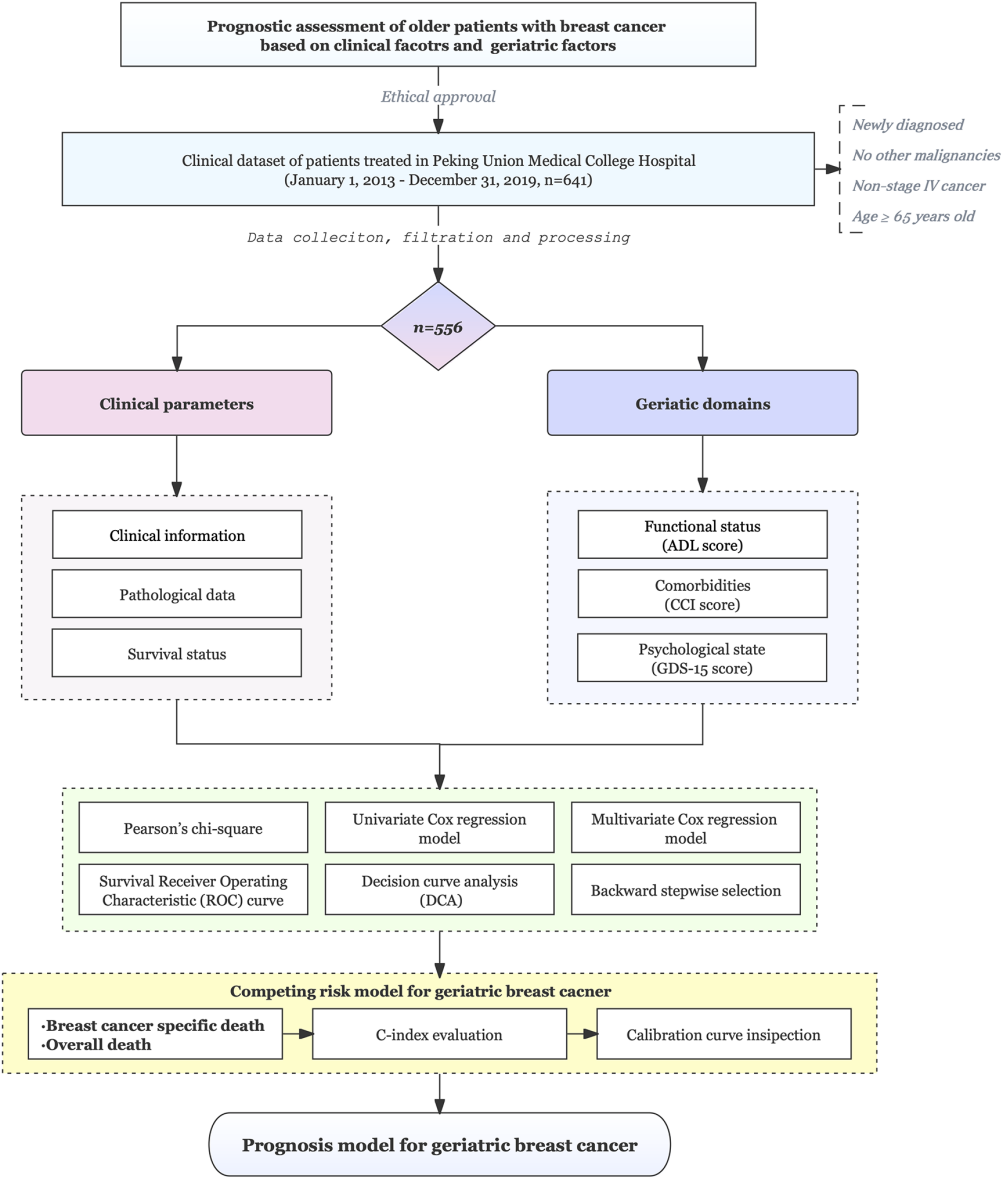
Flowchart of the study. This study included 556 older patients (≥65 years) who underwent breast surgery at a cancer center and the patients were followed for a median of 5.8 years.

The design and procedures of this study are shown in a flowchart (**Figure 1**). Elderly patients (≥65 years) undergoing breast surgery at our cancer unit were enrolled. Of the 641 eligible patients, 556 were enrolled. **Table 1** shows the patient characteristics. Of 556 patients, 172 (31%) were diagnosed with cancer at age ≥75 years. The most common subtype of cancer was luminal B and the most common stage of the tumors was stage I. Significant differences in all-cause mortality (12.8% and 29.7% for <75 years and ≥75 years, respectively) were observed between the two different age groups during 76 months of follow-up. Breast-specific mortality (8.1% vs. 9.3%) was not significantly different between the two groups. The general distribution of patient characteristics, including geriatric assessment domains, were compared (**Table 1**). Functional status differed significantly between the two groups but did not differ significantly with respect to the distribution of intrinsic molecular subgroups, tumor stage, nodal stage, comorbidities, or psychological status.

**Table 1:** Patient characteristics and geriatric assessment domains.

	<75y	≥75y	*P* value
Total number of patients	383(69.0)	172(31.0)	
All-cause mortality	49(12.8)	51(29.7)	**<0.001**
Breast cancer specific mortality	31(8.1)	16(9.3)	0.636
Intrinsic Molecular Subtype			0.578^#^
Luminal A	88(23.0)	33(19.2)	
Luminal B	159(41.5)	79(45.9)	
HER-2 enriched	23(6.0)	8(4.7)	
Basal-like	47(12.3)	18(10.5)	
Unknown	20(5.2)	12(7.0)	
Tumor Stage			0.593^^^
0	46(12.0)	23(13.4)	
I	191(49.9)	90(52.3)	
II	134(35.0)	51(29.7)	
III	9(2.3)	6(3.5)	
unknown	3(0.8)	2(1.2)	
LN Stage			0.114^^^
0	97(25.3)	42(24.4)	
I	36(9.4)	10(5.8)	
II	14(3.7)	12(7.0)	
III	26(6.8)	7(4.1)	
unknown	210(54.8)	101(58.7)	
Functional Status (ADL)			**<0.001^^^**
normal	346(90.3)	128(74.4)	
abnormal	33(8.6)	34(19.8)	
unknown	4(1.0)	10(5.8)	
Comorbidities(CCI score)			0.749
None to Mild	279(72.8)	123(71.5)	
Severe	104(27.2)	49(28.5)	
Psychological state			0.342^^^
Normal	361(94.3)	159(92.4)	
Abnormal	19(5.0)	12(7.0)	
unknown	3(0.8)	1(0.6)	

^#^Pearson’s Chi-squared test without CIS and the unknown cases; ^^^Pearson’s Chi-squared test without the unknown cases. The bold values indicated significance of statistical analysis.

### 3.2 Univariate analysis for GA and clinical parameters

**Figure 2:**
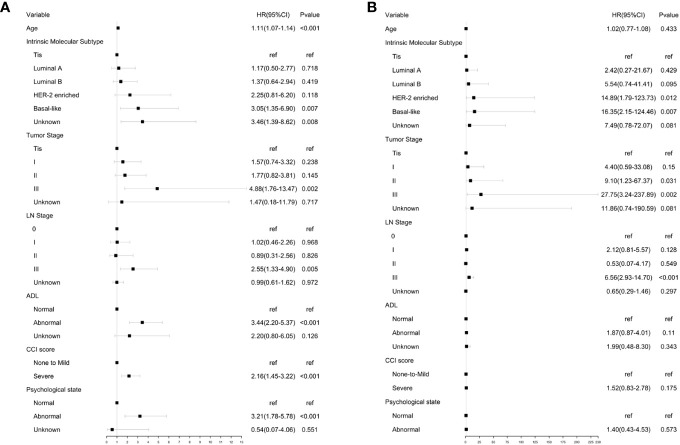
Univariate analysis of OA and BCSS. **(A)** Univariate analysis of OS using GA and clinical parameters. **(B)** Univariate analysis of BCSS using GA and clinical parameters.

A Cox proportional hazards model was constructed for univariate analysis of OS using GA and clinical parameters (**Figure 2A**). Univariate analysis showed that all selected geriatric and clinical parameters were significantly associated with OS. According to the forest plot of univariate analysis, the overall death rate increased with age (HR, 1.11; 95% CI, 1.07 to 1.14), intrinsic molecular subtype (HR for Basal-like, 3.05; 95% CI, 1.35 to 6.90), tumor stage (HR for stage III, 4.88; 95% CI, 1.76 to 13.47), lymph node stage (HR for stage III, 2.55; 95% CI, 1.33 to 4.90), abnormal functional status (HR, 3.44; 95% CI, 2.20 to 5.37), severe comorbidities (HR, 2.16; 95% CI, 1.45 to 3.22), and abnormal psychological state (HR, 3.21; 95% CI, 1.78 to 5.78).

A Cox proportional hazards model was constructed for univariate analysis of BCSS using GA and clinical parameters (**Figure 2B**). Univariate analysis revealed that all clinical parameters were significantly associated with BCSS. The results showed that the risk of breast cancer-associated mortality increased with intrinsic molecular subtype (HR for HER-2 enriched subtype, 14.89; 95% CI, 1.79 to 123.73. HR for basal-like subtype, 16.35; 95% CI, 2.15 to 124.46), tumor stage (HR for stage III, 27.75; 95% CI, 3.24 to 237.89), and lymph node stage (HR for stage III, 6.56; 95% CI, 2.93 to 14.70). No significant differences were observed for BCSS in patients with different functional status (HR:1.87, 95% CI:0.87-4.01), CCI score (HR, 1.52; 95% CI, 0.83 to 2.78), and psychological state (HR, 1.40; 95% CI, 0.43 to 4.53).

### 3.3 Multivariate analysis for GA and clinical parameters

**Figure 3:**
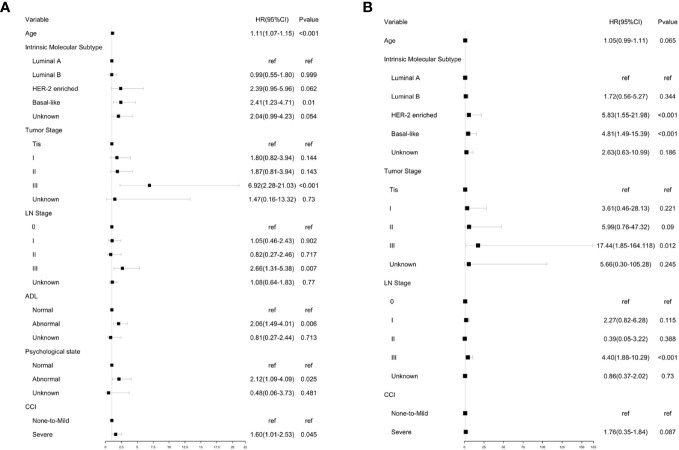
Multivariate analysis of OA and BCSS. **(A)** Multivariate analysis of OS using GA and clinical parameters. **(B)** Multivariate analysis of BCSS using GA and clinical parameters. Multivariate analysis of OS.

Based on the results of univariate analysis, cox proportional hazards models were constructed for multivariate analysis of OS (**Figure 3A**). Similarly, multivariate analysis of BCSS was also performed (**Figure 3B**). Although comorbidities were not significantly associated with BCSS in univariate analysis, they were included in multivariate analysis because coexisting conditions may affect breast cancer management.

Multivariate analysis showed that age, intrinsic molecular subtype, tumor stage, lymph node stage, functional status, comorbidities, and psychological state significantly affected OS in an independent manner. The results showed that patients with Basal-like subtype (HR, 2.41; 95% CI, 1.23 to 4.71), tumor stage III (HR, 6.92; 95% CI, 2.28 to 21.03), lymph node stage III (HR, 2.66; 95% CI, 1.31 to 5.38), abnormal physical status (HR, 2.06; 95% CI, 1.49 to 4.01), abnormal psychological state (HR, 2.12; 95% CI, 1.09 to 4.09), and severe comorbidities (HR, 1.60; 95% CI, 1.01 to 2.53) tended to have shorter OS. It was also noted that the risk of all-cause mortality increased with age (HR, 1.11; 95% CI, 1.07 to 1.15).

Multivariate analysis showed that the intrinsic molecular subtype, tumor stage, and lymph node stage were independent prognostic parameters. The result showed that patients with HER-2 enriched subtype (HR, 5.83; 95% CI, 1.55 to 21.98) and Basal-like subtype (HR, 4.81; 95% CI, 1.49 to 15.39) tended to have shorter BCSS. Compared to other tumor stages, stage III was significantly associated with higher breast cancer specific mortality rate (HR, 17.44, 95% CI:1.85‐164.118). Likewise, patients with lymph node stage III showed a higher risk of breast cancer specific mortality (HR:4.40, 95% CI:1.88‐10.29). However, severe comorbidities did not show a significant correlation with BCSS.

### 3.4 Developing prognostic model for OS

**Figure 4:**
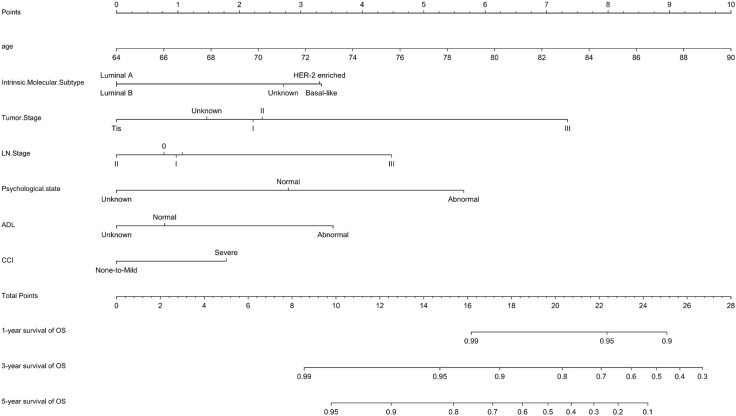
Nomogram for OS. A nomogram was developed based on the multivariate Cox regression analysis to predict the 1-year, 3-year, and 5-year OS.

**Figure 5:**
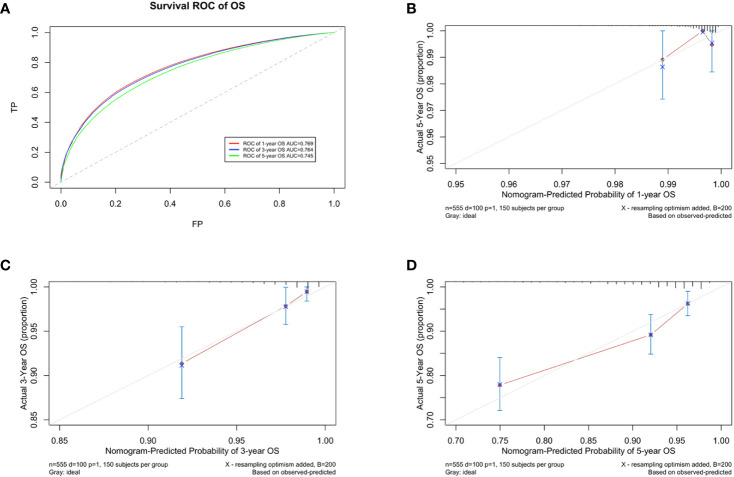
Developing parameters of the nomogram for OS. **(A)** ROC plot for 1-year OS. The AUC of the ROC plot was 0.769, indicating predictive accuracy. **(B)** Calibration plots for 1-year OS. **(C)** Calibration plots for 3-year OS. **(D)** Calibration plots for 5-year OS. The calibration plots showed agreement between the actual and predicted probabilities.

A prognostic model for OS was developed based on the multivariate Cox regression analysis. A nomogram including age, intrinsic molecular subtype, tumor stage, lymph node stage, functional status, comorbidities, and psychological state was constructed to predict the 1-, 3-, and 5-year OS by summing each factor score to calculate the total score (**Figure 4**). The predicted C-index of the OS prognostic model was 0.748 (95% CI:0.695-0.800). The 1-year OS prediction (AUC=0.769), 3-year OS prediction (AUC = 0.764), and 5-year OS prediction (AUC = 0.745) are shown in the ROC plot (**Figure 5A**). The calibration plot revealed good predictive accuracy between the actual and predicted probabilities for 1-year, 3-year and 5- years OS (**Figures 5B–D**, respectively).

**Figure 6:**
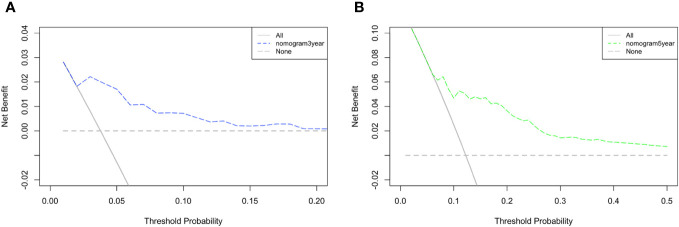
DCA of the nomogram. The DCA indicated that the nomogram had potential for clinical utility of **(A)** 3-year and **(B)** 5-year OS.

A DCA of the nomogram was conducted, which indicated that the nomogram had potential for clinical utility. The DCA indicated that when the threshold probability for 3-year and 5- years OS (**Figures 6A, B**, respectively) were within the range (2.5%-17% and 8%–50%, respectively), the nomogram added more net benefit than “all or none” strategy. Enough number of events did not occur to support the DCA of the nomogram for 1-year.

### 3.5 Developing prognostic model for BCSS

**Figure 7:**
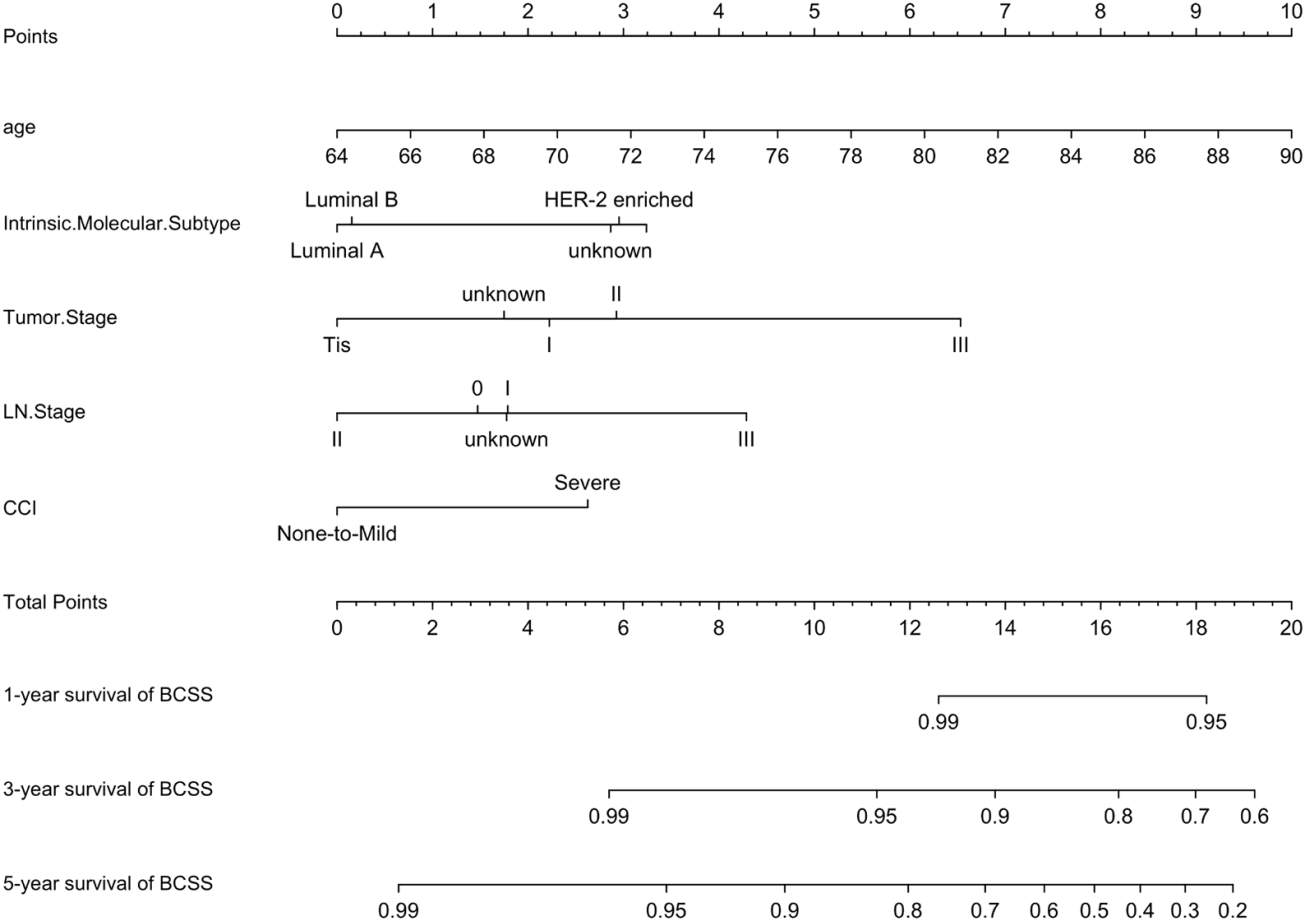
Nomogram for BCSS. A nomogram was developed based on the multivariate Cox regression analysis to predict the 1-year, 3-year, and 5-year BCSS.

A prognostic model for BCSS was developed based on the multivariate Cox regression analysis. A nomogram, including age, intrinsic molecular subtype, tumor stage, lymph node stage, and comorbidities, was built to predict the 1-, 3- and 5-year BCSS by summing each factor score to calculate the total score (**Figure 7**).

**Figure 8:**
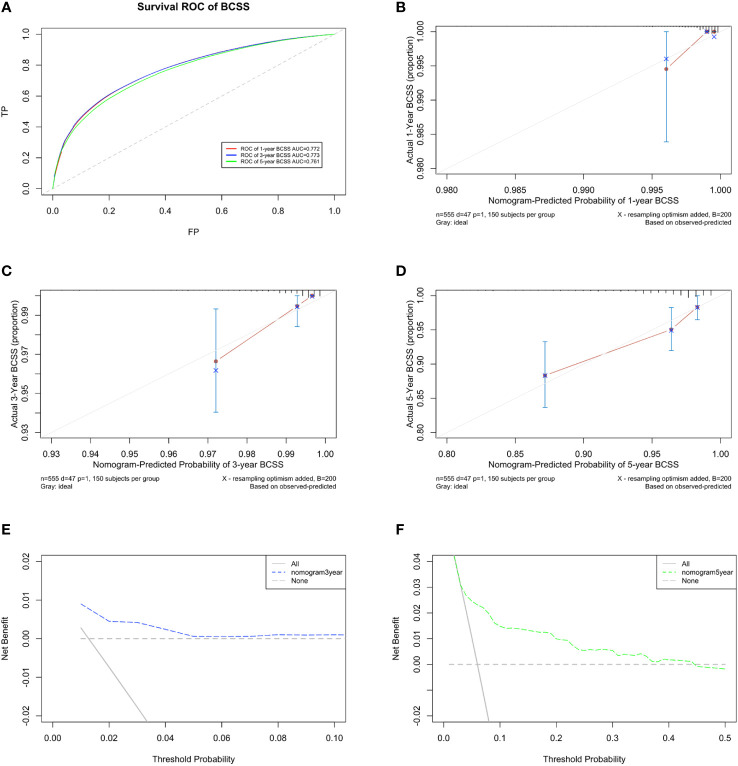
Developing parameters of the nomogram for BCSS. **(A)** ROC plot for 1-year BCSS. The AUC of the ROC plot was 0.772, indicating predictive accuracy. **(B)** Calibration plots for 1-year BCSS. **(C)** Calibration plots for 3-year BCSS. **(D)** Calibration plots for 5-year BCSS. The calibration plots showed agreement between the actual and predicted probabilities. The calibration plots showed agreement between the actual and predicted probabilities. **(E)** DCA of the nomogram for 3-year and **(F)** 5-year BCSS. The DCA indicated that the nomogram had potential for clinical utility.

The predicted C-index of the BCSS prognostic model was 0.760 (95% CI:0.753-0.834). The 1-year (AUC=0.772), 3-year (AUC = 0.773), and 5-year BCSS risk predictions (AUC = 0.761) are shown in the ROC plot (**Figure 8A**). The calibration plot revealed good predictive accuracy between the actual and predicted probabilities for 1year, 3-year and 5- year BCSS. (**Figures 8B–D**, respectively).

DCA of the BCSS nomogram was also performed, indicating potential clinical utility of the nomogram. The DCA indicated that the nomogram added more net benefit than the “all or none” strategy when the threshold probabilities for 3-year and 5-year BCSS (**Figures 8E, F**) were in the range (1.5%-4.5% and 5%-45%, respectively).Enough number of events did not occur to support the DCA of the nomogram for 1-year.

## 4 Discussion

Tumor stage, lymph node stage, and intrinsic molecular subtypes are strongly associated with breast cancer prognosis (19, 20). Consistent results were also attained from our cohort study of senior patients with breast cancer, wherein both univariate and multifactorial analyses demonstrated significance in OS and BCSS analyses. Therefore, these three clinical domains were included in the nomograms of both OS and BCSS.

Our study also found that half of the risk of breast cancer-associated mortality in older patients is due to breast cancer, whereas the other half stems from factors such as aging and comorbidities. This is also in concordance with the previous findings, which suggests that it is not enough to only consider clinical oncological indicators when assessing the prognostic risk of older patients with breast cancer and that the importance of geriatric assessment in clinical decision-making should be fully appreciated (21).

GA helps understand the heterogeneity of older patients, which is essential to guide treatment planning for older patients with breast cancer and is recommended by the 2012 edition of the International Consensus on the Treatment of Elderly Breast Cancer (22). However, it is rare to find published studies that incorporate the assessment of GA in the treatment of elderly patients with breast cancer, and no such studies have been reported in China. To the best of our knowledge, this is the first study ever to integrate GA into the prognostic models in the field of surgical oncology in China. The analysis in this study showed that all three GA domains and three clinical parameters, as well as age, had prognostic value in the survival analysis of this group of older cancer patients.

Functional status has been addressed in several previous studies of solid tumors in the older population, all of which were consistent with the conclusion that it is related to survival (12, 23–25). Similar findings were found in our study, wherein the effect of functional status on OS was significant in univariate analysis and was also an important prognostic factor in multivariate analysis. However, there was no significant correlation with BCSS. This could be interpreted as poor functional status or possible undertreatment due to poor function, which did not have a significant impact on breast cancer treatment.

Comorbidity is an essential factor for GA and has been consistently suggested in previous studies to be associated with decreased survival in older patients with different types of cancers (26–28). These studies showed that the presence of comorbidities reduces OS and tumor-related survival, and that the latter may be due to the fact that the presence of comorbidities causes undertreatment of specific tumors, thus reducing tumor-related survival (29). In our study, this risk was present only for all-cause mortality and was not significantly associated with BCSS. A possible explanation may be related to the tumor characteristics. Most breast cancers in the older population are hormone receptor expression-positive and the side effects of endocrine therapy are relatively less, which means that the interactions of comorbidities have little impact. Nevertheless, we still identified comorbidities as important prognostic factors in our BCSS prognostic model based on previous relevant literature.

Psychological state is also a significant factor affecting survival in cancer patients. Moreover, it has been shown to be associated with an increased risk of death in patients (30). This was also observed in our patient cohort, where psychological state significantly influenced OS, but was not significantly associated with BCSS. Therefore, psychological state was identified as a significant prognostic factor only in the OS prognostic model. Breast cancer has a relatively longer survival period than other types of cancers; therefore, psychological problems appear more prominent during the relatively long survival period (31). Our study suggests that the psychological state of older patients with breast cancer should be taken seriously, and early intervention should be performed if problems arise.

To the best of our knowledge, this is the first breast cancer nomogram based on selected GA and clinical prognostic parameters, which would allow a simplified and personalized prognostic process. The developed nomograms can predict the overall risk of death and the risk of breast cancer-related death in older patients over a 5-year period with relatively good accuracy. It is our expectation that this will be translated for use in clinical patients, thereby providing information to support clinical decision making for the treatment of patients. It is also expected that our study will trigger concern for older breast cancer patients and that these geriatric factors will be fully considered in more clinical studies and practices.

Our study had a few limitations. Since the data from a single-center was used retrospectively, there may have been a case selection bias. In addition, since we belong to the breast surgery department, the cases included in the study were those of older patients admitted to the surgical ward for surgical procedures. Thus, the patients included in the study were relatively less frail, which may have distorted the results. Furthermore, for increased accuracy, we utilize ADL assessment instead of ECOG. Although ECOG may have higher reproducibility and convenience in clinical practice. In the next phase of our study, we aim to collect both outpatient and inpatient data, including those of older patients who cannot tolerate surgery due to underlying diseases, to make the data more informative and realistic.

## Data availability statement

The original contributions presented in the study are included in the article/supplementary material. Further inquiries can be directed to the corresponding author.

## Ethics statement

The studies involving humans were approved by Institutional Review Board of Peking Union Medical College Hospital (ZS-2682). The studies were conducted in accordance with the local legislation and institutional requirements. The participants provided their written informed consent to participate in this study.

## Author contributions

YL: methodology, project administration, writing. YS: writing, reviewing and editing. YinX: writing and editing. CW: reviewing and editing. YalX: reviewing and editing. XH: reviewing and editing. QS: Supervision, conceptualization, and funding acquisition.
